# Nanomedicine for Ischemic Stroke

**DOI:** 10.3390/ijms21207600

**Published:** 2020-10-14

**Authors:** Xinyue Dong, Jin Gao, Yujie Su, Zhenjia Wang

**Affiliations:** Department of Pharmaceutical Sciences, College of Pharmacy and Pharmaceutical Sciences, Washington State University, Spokane, WA 99202, USA; xinyue.dong@wsu.edu (X.D.); jin.gao3@wsu.edu (J.G.); yujie.su@wsu.edu (Y.S.)

**Keywords:** ischemic stroke, blood brain barrier, nanoparticle-based drug delivery, brain targeting

## Abstract

Stroke is a severe brain disease leading to disability and death. Ischemic stroke dominates in stroke cases, and there are no effective therapies in clinic, partly due to the challenges in delivering therapeutics to ischemic sites in the brain. This review is focused on the current knowledge of pathogenesis in ischemic stroke, and its potential therapies and diagnosis. Furthermore, we present recent advances in developments of nanoparticle-based therapeutics for improved treatment of ischemic stroke using polymeric NPs, liposomes and cell-derived nanovesicles. We also address several critical questions in ischemic stroke, such as understanding how nanoparticles cross the blood brain barrier and developing in vivo imaging technologies to address this critical question. Finally, we discuss new opportunities in developing novel therapeutics by targeting activated brain endothelium and inflammatory neutrophils to improve the current therapies for ischemic stroke.

## 1. Introduction

Stroke is an unexpected and acute brain disease. It is reported that one of nineteen deaths is related to stroke in the United States, and the mortality rate of stroke is as high as 30% [1]. Stroke is defined by a condition caused by a hemorrhage or occlusion of cerebral blood vessels. Lacking of blood flow in the brain causes dysfunctions of brain cells, oxidative stress, and neurological damage [2]. The symptoms of stroke include numbness, confusion, and aphasia, and those signs are related to injured areas in the brain [3]. Since 87% of strokes are related to ischemia in the brain and 13% are involved with the hemorrhage, this review will focus on discussing how nanotechnology improves therapies and diagnosis of ischemia stroke.

Cerebral ischemia initiates a cascade of pathological processes, eventually causing neuron death. Reperfusion is a clinical method to restore the blood flow in the brain by administration of tissue plasminogen activator (t-PA) or mechanical thrombectomy (MT) for treatment of ischemic stroke [4]. However, reperfusion often leads to tissue damage because oxygen influx of reperfusion generates reactive oxygen species (ROS), initiating inflammatory responses including cytokine production and leukocyte infiltration [5]. Many neuroprotectants have been developed to alleviate reperfusion-induced injury, but none of them were clinically approved. There are several reasons for this failure: (1) ineffective drug delivery into the brain because of blood brain barrier (BBB), (2) drugs with short circulation times, poor stability, and toxicity, (3) difficulty in choosing right drugs and doses due to the heterogenicity of stroke (e.g., disease locations and severity).

Recently, nanotechnology emerges as innovative tools in drug delivery and diagnosis to treat a wide range of diseases, such as cancer and inflammatory disorders [6,7,8,9,10,11,12,13]. In the case of ischemic stroke, nanoparticles could possibly deliver therapeutics across BBB, prolong the drug circulation, and increase the drug accumulation at diseased sites. In addition, nanoparticles can be utilized as innovative diagnostic systems to detect several biomarkers (such as ROS and neurotransmitters) in the brain for early-stage stroke diagnosis. In this review, we firstly describe the physiopathology of ischemic stroke and current limitations in therapy and diagnosis. Then, we discuss how nanotechnology offers opportunities in treating ischemic stroke, highlighting the recent progress on delivering neuroprotective agents, anti-inflammatory drugs, and small interfering RNA (siRNA) to alleviate ischemic injuries. We also review the applications of nanomaterials in stroke diagnosis. Finally, we describe new opportunities in translating nanoparticle-based therapies to clinic in the prevention and treatment of ischemic stroke.

## 2. Pathology of Stroke and Current Therapies

### 2.1. Molecular Mechanisms for Pathology in Stroke

**Figure 1:**
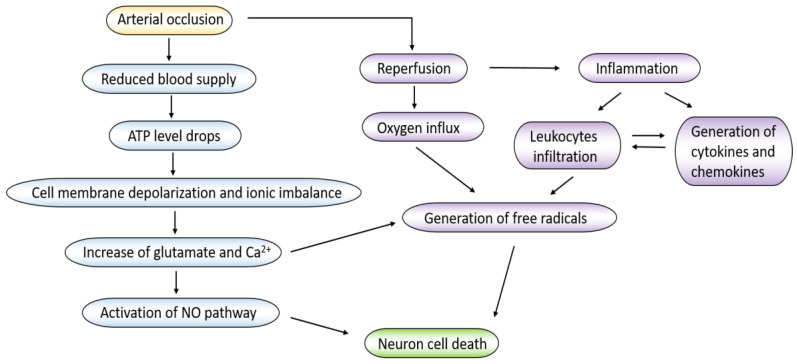
The cellular process and mechanism of ischemic stroke and the ischemic injury cascade.

Acute ischemic stroke and subsequent reperfusion cause a series of pathophysiological changes including neuronal damage, oxidative stress, local inflammation, and loss of blood brain barrier (BBB) integrity [14]. The cellular process and mechanism of ischemic stroke and the ischemic injury cascade is summarized in Figure 1. Herein, we mainly discuss neuronal damage, oxidative stress, and inflammation responses that contribute to the pathogenesis of ischemic stroke. These three events are intertwined, and each can result in brain damage.

#### 2.1.1. Neuronal Damage

Neurons are the major components in brain and are particularly vulnerable to ischemia. Studies have shown that acute cerebral ischemia causes the vasculature infarction, thus leading to neuronal death in rodent models [15]. Due to reduced blood supply in the ischemic brain, adenosine triphosphate (ATP) level drops and lactate acidosis leads to the loss of ionic homeostasis in neurons [14]. Failure to maintain the ion gradients results in depolarization of cell membranes and therefore activating a variety of ion channels such as calcium and sodium channels. Those events also cause the excessive release of glutamate, which initiate more loss of neuronal cells [16]. Glutamate is a major excitatory neurotransmitter in nervous system regulating a variety of excitatory synapses, but high levels of extracellular glutamate can induce the cell death in the late stage. Dead neural cells release damage-associated molecular patterns (DAMPs), which activate the innate and adaptive immune system, initiating inflammatory pathways [17].

#### 2.1.2. Oxidative Stress

Oxidative stress is involved with the production of reactive oxygen species (ROS), which activates apoptosis, necrosis, and autophagy pathways during ischemic stroke [18]. Oxidative stress also reduces the bioavailability of nitric oxide (NO) in endothelial cells. Nitric oxide is a potent chemical that inhibits platelet aggregation and leukocyte adhesion [19]. The loss of NO will exacerbate the coagulation, which aggravates the ischemic insult, leading to more blood flow reduction in the brain. In addition, cells under ischemia can increase the levels of calcium, sodium, and adenosine diphosphate (ADP) that produce oxygen species in mitochondria. Reperfusion is a commonly used method to treat ischemic stroke, but this also produces more ROS to cause the secondary damage in the brain. Furthermore, ROS damages lipids, proteins, and nucleic acid via lipid peroxidation, protein oxidation, and DNA fragmentation and leads to further cell death [20].

#### 2.1.3. Inflammation Response

Inflammation response is an important event in ischemic stroke and the subsequent reperfusion. Ischemia and reperfusion activate immune system to respond to tissue injury including activation of endothelium and leukocytes. Specifically, the immune response is involved with the intravascular and parenchymal damage triggered by the interruption of blood supply. In other words, the immune system plays a central role in regulating the outcomes of stroke patients [21]. For the immune response, adhesion molecules on endothelial cells are upregulated during ischemia. For example, P-selectin was highly expressed when the mouse brain was damaged. P-selectin is a major adhesion molecule for the binding of platelets to endothelial cells, and this interaction causes proinflammatory signals [22]. ICAM-1 is another intercellular adhesion molecule and is upregulated during inflammation response. A study showed that knockdown of intercellular adhesion molecule-1 (ICAM-1) gene in mice reduced the infarction of the brain, suggesting that inflammation response plays a major role in pathogenesis of stroke [23]. 

Innate immune cells are major components during inflammation response because they sense endogenous danger signals through several receptors such as toll-like receptors (TLRs), retinoic acid-inducible gene (RIG)-1-like receptors, nucleotide oligomerization domain (NOD)-like receptors, C-type lectin receptors, and absent in melanoma (AIM2)-like receptors [24]. These receptors activate downstream signaling pathways, such as nuclear factor-κB (NF-κB), mitogen-activated protein kinase, and type 1 interferon pathways. These pathways upregulate proinflammatory cytokines, chemokines, and oxidative metabolites. The products including tumor necrosis factor alpha (TNF-α), interleukin-1β (IL-1β), IL-6, IL-18, and NO activate endothelial cells and astrocytes, and also act on leukocytes for their tissue infiltration in the brain, thus causing more tissue damage. Clinical studies found that in stroke patients, the increase of cytokines appeared in the cerebrospinal fluid and blood and this feature is correlated to patient survival [25]. In addition, the increased infiltration of leukocytes caused by the formation of fibrin and cytokines contributes to further tissue damage. Ischemia and reperfusion activate macrophages in the perivascular space. Activated macrophages release proinflammatory cytokines, which contribute to the expression of adhesion molecules in endothelium, promoting more leukocyte infiltration (especially neutrophils) to cause the BBB damage [26]. The large infiltration of neutrophils in a short period causes the release of proteases that can further destroy the BBB integrity and exacerbate oxidative stress, leading to severe brain damage.

### 2.2. Blood Brain Barrier in Ischemic Stroke

Blood brain barrier (BBB) plays an important role in ischemia/reperfusion (I/R). During ischemic stroke, there are two phases of BBB breaking. In the early phase of reperfusion (6 h), endothelial transcytosis dominates in increased permeability of BBB, and it may be associated with endothelial vesicle transport [27]. In the later phase, BBB is disrupted because of the loss of integrity of endothelial tight junctions (TJs). Studies showed that the structural changes of TJs occurred at 48–58 h after middle cerebral artery occlusion (MCAO) in mice [28], and these changes were associated with the degradation of TJs by matrix metalloproteinases (MMPs) [29]. MMPs also degraded extracellular matrix proteins of basal membrane of BBB, such as type IV collagen [30]. 

The immune response also causes the loss of BBB integrity, leading to cell death in stroke [31]. Microglia are macrophages existing in the brain, and they become activated in response to ischemic stroke, which cause the production of ROS, cytokines (e.g., TNF-α, IL-1β, and IL-6) and chemokines (e.g., macrophage inflammatory proteins-1alpha (MIP-1α)/CCL3, monocyte chemoattractant protein-1 (MCP-1)/CCL-2 and chemokine (C-X-C motif) ligand-1 (CXCL-1)). These inflammatory products act on endothelial cells to activate the NF-κB pathway for expression of adhesion molecules, such as vascular cell adhesion protein (VCAM), ICAM-1, and P-selectin. These molecules support leukocyte recruitment to invade brain parenchymal tissues [32], thus increasing the brain permeability [33].

Blood brain barrier maintains the brain homeostasis, but ischemic stroke disrupts this barrier, resulting in increased vascular permeability. As we discussed, the permeability is regulated by transcellular pathways in the early stage and the breaking TJs in the later stage [28]. Therapeutics delivery systems to ischemic stroke lesions can be designed based on its pathogenesis. 

### 2.3. Current Treatment Options for Ischemic Stroke

**Figure 2:**
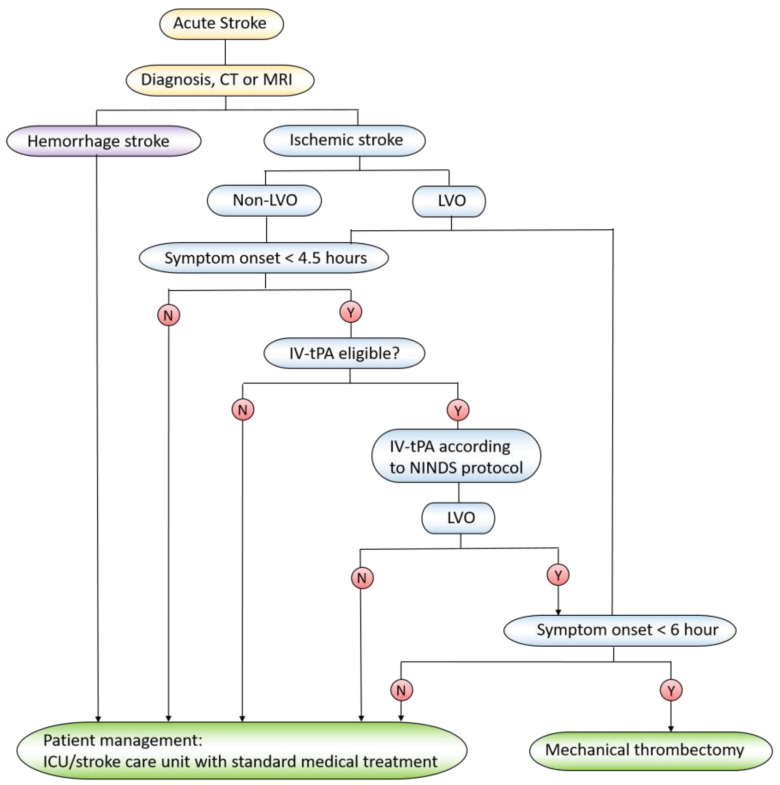
The procedure to treat stroke patients. CT: computed tomography; MRI: magnetic resonance imaging; LVO: large vessel occlusion; IV-tPA: intravenous injection of trans-plasminogen activator; ICU: intensive care unit; NIHSS: National Institutes of Health Stroke Scale; Y: yes; N: no.

Treating ischemic stroke is to restore blood flow to the brain as soon as possible, and reperfusion is a major tool to bring the blood back to the brain. The clinical methods include intravenous administration of tissue plasminogen activator (tPA) and mechanical thrombectomy (MT) [34]. Tissue plasminogen activator (tPA) is the gold standard treatment for ischemic stroke. The treatment window using tPA is in 4.5 h when patients show the stroke symptom, and the outcomes dramatically decrease beyond 4.5 h [35]. However, tPA treatment may potentially cause the intracerebral hemorrhage and lead to more mortality [36]. When patients appear with a large artery occlusion (the clot burden is high) or tPA treatment is beyond the best time window, the MT surgery is an alternative strategy to treat ischemic stroke. In MT surgery, a microcatheter is inserted to clotting blood vessels to remove the clots. The treatment procedure for stroke patients is summarized in Figure 2.

Except for the reperfusion, supportive care is also very important. For example, 25% of patients may show neurological deficiency in 24–48 h, and care is needed to prevent further brain damage [37]. Although stroke and its pathogenesis have been investigated for many years, there are no pharmacological agents available in clinic to effectively treat ischemic stroke [38,39].

There are several drugs that have been tested to target inflammatory pathways in ischemic stroke. NXY-059 is a scavenger of free radicals and shows the neuroprotective effect in a pre-clinical stroke model [40]. However, NXY-059 was less effective in Stroke–Acute Ischemic NXY Treatment II (SAINT II) trial [41]. The failure may be due to the high water solubility of NXY-059 that has low permeability to BBB. Therefore, developing new drug delivery approaches are needed to solve the current issues on treating ischemic stroke. 

## 3. Nanomedicine in Stroke Treatment

**Figure 3:**
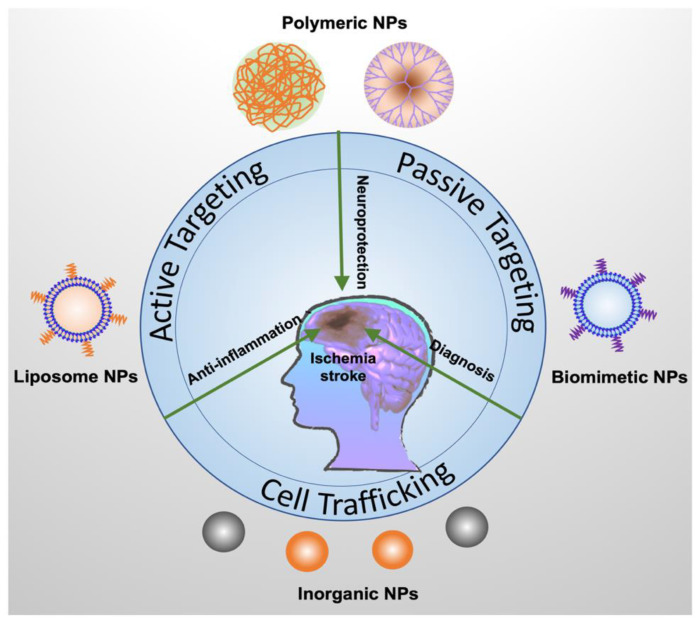
Nanoparticle-based delivery platforms for delivery of neuroprotectants, anti-inflammation reagents, and imaging probes for ischemic stroke therapy and diagnostics. Polymeric NPs include PLGA (poly(lactic-co-glycolic acid) NPs, chitosan NPs, and PAMAN dendrimer. Liposomes are made of a lipid bilayer and are loaded with therapeutics agents. Inorganic NPs include metallic NPs (such as platinum), ceria NPs, and Fe_2_O_3_ NPs. Biomimetic NPs are new drug delivery platforms generated from cell membrane vesicles. Each type of NPs has been discussed in the manuscript.

There are several challenges in treating ischemic stroke. Most therapeutics have short blood circulation and cannot easily cross the blood brain barrier to reach the ischemic tissues. In addition, controlling drug release and developing novel drug delivery vehicles are important factors to improve therapies of ischemic stroke. Nanotechnology is used to design nanoscale materials that can be applied in biomedical applications. Nanoparticles with the sizes of 50–200 nm are generated and can encapsulate drugs to increase drug water solubility and tissue targeting. Nanoparticles are smaller than a cell and larger than small molecules; thus, nanoparticles are excellent carriers to deliver therapeutics (drugs) and control their release for improved therapies in a wide range of diseases [42,43,44]. Nanoparticle-based drug delivery systems may solve the current challenges in ischemic stroke treatment. In this section, we present several nanoparticle-drug delivery systems used in treating ischemic stroke (Figure 3).

### 3.1. Delivery of Neuroprotectants

Early recanalization (thrombolysis) and neuroprotection are the two major steps for stroke patients [45]. Neuroprotection is an important strategy to protect the neurological damage from ischemic stroke. There are several approaches including reduced immune cell adhesion, blocking of proinflammatory cytokines, decreased lipid peroxidation, and decreased cell apoptosis. Many drugs, such as adenosine [46], have been developed and approved by the FDA to treat myocardial ischemia. However, they failed to be used in treating cerebral I/R injury [47] because they have the short plasma half-life time and lack of permeability to BBB. To address this issue, nanoparticle drug delivery systems have been developed to deliver drugs to cross BBB. The applications of nanoparticle-drug delivery systems have been summarized in Table 1. 

#### 3.1.1. Liposomes as Delivery Platforms

Liposomes are the first generation of nanocarriers for drug delivery used in academia and pharmaceutical industries [71]. Liposomes are made of biocompatible and biodegradable molecules that can protect drugs from enzymatic degradation and prevent drug clearance in blood. 

Ishii and co-workers developed asialo-erythropoietin (AEPO)-modified PEGylated liposomes (AEPO-liposomes) to treat cerebral I/R injury [48]. Asialo-EPO (AEPO) is a neuroprotective agent that binds to EPO receptor (EPOR) on neuronal cells and activates MAPK and PI3K/Akt pathways for improved outcome of cerebral stroke. The results showed that AEPO-liposomes accumulated in the ischemic lesion for more than 24 h after injection, decreased infarct lesions, reduced the neuronal apoptosis, and ameliorated cerebral I/R injury in rats. 

ZL006 is another neuroprotectant that can selectively block the ischemia-induced binding between nNOS (neuronal nitric oxide synthase) and PSD-95 (a scaffolding protein) to prevent ischemic damage [72]. However, this drug is limited to cross the BBB. To solve this problem, HAIYPRH (T7), a peptide for targeting transferrin receptor (TfR), was conjugated to PEGylated liposomes (T7-P-LPs) loaded with ZL006 (T7-P-LPs/ZL006), and the liposomes mediated the transport of ZL006 across BBB in the ischemic stroke model [49]. The results in ex vivo fluorescence imaging and particle biodistribution studies suggested that T7 enhanced the transport of liposomes across the BBB. T7-PLPs/ZL006 also significantly reduced infarct volume and prevented neurological deficit in the rat ischemic stroke model compared to free ZL006. The same group developed another dual targeting delivery system using T7 peptide and stroke homing peptide (SHp, CLEVSRKNC)-conjugated liposome (T7&SHp-P-LPs/ZL006) and demonstrated that this system can penetrate BBB to deliver ZL006 to the ischemia brain tissue. Administration of T7&SHp-P-LPs/ZL006 decreased the infarction sizes in the brain and ameliorated neurological damage [50].

Basic fibroblast growth factor (bFGF) has demonstrated the neuroprotective effect in acute stroke; however, bFGF encounters the same issue that it cannot cross the BBB. To overcome this problem, liposomes were developed to deliver bFGF (bFGF-NL) to the brain in a rat model of cerebral I/R. After treatment with bFGF-NL, in three consecutive days, mice showed improved neurological function, reduced infarct volume, and decreased nuclear fragmentation of neurons. The therapeutic effect is associated with PI3K/Akt activation by bFGF-NL [51]. 

To address the problem of narrow therapeutic time window of tPA (4.5 h), Fukuta et al. combined tPA and liposomal fasudil (fasudil-Lip) and examined whether they can increase the benefit of tPA to treat cerebral I/R. The results showed that the combined therapy increased neuroprotective effects compared to the administration of tPA or fasudil alone. In addition, the study indicated that administration of fasudil-Lip decreased the cerebral hemorrhage and extended the therapeutic time window of tPA compared to treatment with tPA [52].

To determine when drugs should be administered after reperfusion surgery, Al-Ahmady et al. selected a liposomal formulation made from HSPC:CHOL:DSPE-PEG2000 because PEGylated-liposomes have a long blood circulation. In the study, liposomes were intravenously administered at 0.5 or 48 h after reperfusion. Transmission electronic microscopy, histological, and immunostaining were performed, and the results indicated a higher accumulation of liposomes in cerebral ischemic mice than that in healthy mice. The possible mechanism was that the increased accumulation of liposomes in brain in the early phase (0.5 h) was due to transcellular transport. However, at 48 h after stroke, the liposome deposition was associated with both transcellular and paracellular pathways [53]. This is an interesting study, but it is needed to investigate the time course of liposome accumulation in ischemic stroke tissues to determine the therapeutic delivery time to improve the treatment of stroke.

#### 3.1.2. Polymeric Nanoparticles as Delivery Platforms

Biodegradable polymeric NPs have also been developed as carriers for drug delivery to treat neurological diseases because they are not toxic and biocompatible, and they have the sustained-release feature [73]. Liu and co-workers developed tanshinone IIA PEGylated nanoparticles (CBSA-PEG-TIIA-NPs) conjugated with cationic bovine serum albumin (CBSA) to target negatively-charged lumens of brain microvessels [54]. The study demonstrated that NPs delivered tanshinone IIA into the rat brain in the cerebral I/R injury model, significantly suppressing the inflammation response. In addition, NPs reduced the infarcted volume and prevented the neuronal apoptosis. In another study, the same group investigated the mechanism of therapeutic effects using CBSA-PEG-TIIA-NPs. They found that NPs decreased neutrophil infiltration and prevented microglial activation. Furthermore, it is shown that prevention of neuronal apoptosis and suppression of inflammatory mediators (such as MMP-9 and COX-2) were regulated by mitogen-activated protein kinases (MAPK) signal pathways [55].

Nanoparticles were also bioengineered with ROS-responsive features that can control drug release in ischemic brain. In a study [56], Lv et al. designed nanoparticles to deliver NR2B9C (a neuroprotectant agent) to treat ischemic stroke. Nanoparticles (named SHp-RBC-NPs) composed of a red blood cell (RBC) membrane as a shell and a polymer nanoparticle with ROS-responsive boronic ester as a core. To target the ischemic brain, a peptide, SHp (CLEVSRKNC), was conjugated on the surface of nanoparticles. Triggered by high levels of ROS in ischemic region, the nanoparticles can control the release of NR2B9C in ischemic brain tissues. Ex vivo brain fluorescence imaging showed that SHp-RBC-NP can target the ischemic brain to significantly prevent neurological damage and reduce the brain infarction size.

3-n-Butylphthalide (dl-NBP) [74] has demonstrated the therapeutic value to treat ischemic stroke in clinic, but it is challenging to deliver it to the ischemic brain. To solve this problem, PEGylated-lipid nanoparticles (PLNs) conjugated with Fas ligand was developed to target the ischemic region of the brain and deliver 3-n-Butylphthalide (dl-NBP). The results are promising as the nanoparticle formulation alleviates the brain neurological injury [57].

Guo et al. designed AMD3100 (a targeting ligand)-conjugated and size-shrinkable nanoparticles (ASNPs) with the features of protease-responsiveness and brain-targeting. The nanoparticles specifically targeted the CXCR4 (C-X-C chemokine receptor type 4)-enriched ischemic brain tissue and penetrated the ischemic brain. Glyburide, a promising anti-stroke drug, was delivered by the nanoparticles, and this system enhanced the therapeutic outcomes in the ischemic stroke model [58]. 

Peptide inhibitors for caspases possess neuroprotection effects, but they also cannot cross the BBB. Based on this problem, Karatas et al. developed chitosan nanospheres loaded with N-benzyloxycarbonyl-Asp(OMe)-Glu(OMe)-Val-Asp(OMe)-fluoromethyl ketone (Z-DEVD-FMK), a caspase-3 inhibitor. Anti-mouse transferrin receptor monoclonal antibody (TfRMAb) was conjugated to polyethylene glycol-coated nanospheres because the antibody can selectively bind to TfR type 1 expressed on cerebral vasculature. Nanospheres suppressed the caspase-3 activity and improved neurological repair after ischemic stroke [59].

#### 3.1.3. Biomimetic Nanoparticles as Delivery Platforms

Cell-derived biomimetic carriers have provided new options for drug delivery due to their advanced targeting abilities and better biosafety than artificial carriers. Shi et al. reported a T7 peptide (a brain targeting peptide)-linked erythrocyte membrane nanovesicles loaded with Mn_3_O_4_ nanoparticles (Mn_3_O_4_@nanoerythrocyte-T7, MNET), and this complex formulation may scavenge free radicals and change hypoxia environments in the ischemic brain. They found that MNET had a long circulation time and were capable of crossing BBB. In subsequent studies, MNET scavenged free radical and oxygen supply to rescue neurons during ischemic phase, and possibly MNET helped to remove oxygen generated by reperfusion [60].

Platelet membrane-derived nanovesicles were loaded with L-arginine, a nitric oxide (NO) donor, and γ-Fe_2_O_3_ magnetic nanoparticles (PAMNs) to achieve dual therapeutic and diagnostic purposes. The PAMNs were able to adhere to the damaged cerebral vessel induced by thrombosis and deliver L-arginine when the external magnetic field was applied. Furthermore, the release of L-arginine at ischemic lesions promoted vasodilation and the thrombosis was disrupted to restore the blood flow. This study showed that PAMNs may be a useful tool for both MRI imaging and targeted therapy for ischemic stroke [61]. 

#### 3.1.4. Inorganic Nanoparticles as ROS Scavengers

Studies have showed that metallic NPs can function as scavengers of ROS. ROS includes superoxide anion, hydrogen peroxide, and hydroxyl radical, and they are generated during ischemic stroke. ROS is involved with oxidative tissue damage that is the mechanism responsible for brain injury in ischemic stroke.

**Figure 4:**
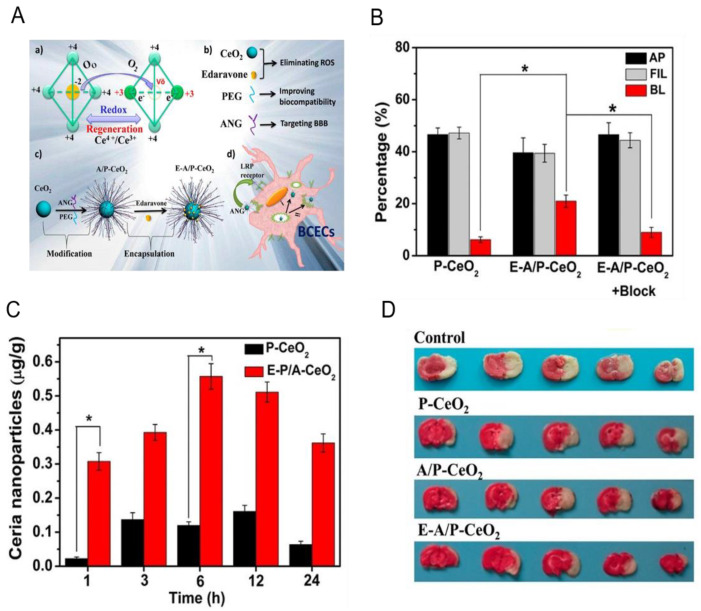
Edaravone-loaded ceria nanoparticles cross the blood brain barrier and protect the brain from ischemic stroke. (**A**) The nanoplatform is comprised of ceria nanoparticle core, PEG shell, and ANG as targeting peptide. (**a**) Transition between cerium(III) and cerium(IV) species in ceria NPs; (**b**) main components of the nanoparticle system; (**c**) synthetic procedure for E-A/P-CeO_2_; (**d**) receptor-mediated (ANG-LRP) endocytosis of E-A/P-CeO_2_. (**B**) Transmigrated amount of P-CeO_2_ and E-A/P-CeO_2_ in in vitro transmigration model. Free ANG was used as the blocking agent in group 3. (**C**) Concentrations (µg Ce/g brain tissue) of ceria nanoparticles in normal brain tissue. The injection dose was 0.5 mg/kg. (**D**) Representative TTC (triphenyl tetrazolium chloride)-stained brain sections after different treatments within 24 h of stroke. Data represent mean ± standard deviation (SD). * indicates *p* < 0.05. Reproduced with permission [63]. Copyright 2018, American Chemical Society.

It is reported that ceria nanoparticles can scavenge free radicals by reversibly reacting with oxygen to Ce^4+^ (oxidized) species from Ce^3+^ (reduced) species [62]. Kim et al. firstly reported that ceria nanoparticles reduced ROS production and prevented cell apoptosis, thus protecting the brain from ischemic damage. Although ceria nanoparticles are effective to scavenge ROS, the integrity of BBB is the barrier to prevent their brain deposition. Bao and coworkers developed ceria nanoparticles (E-A/P-CeO_2_) modified with Angiopep-2 (ANG) and poly(ethylene glycol), which were able to cross BBB via receptor-mediated transcytosis (Figure 4A). In vitro transmigration assay (Figure 4B) showed that E-A/P-CeO_2_ could cross the brain capillary endothelial cells (BCECs) compared to P-CeO_2_ [63]. The BBB crossing capability of E-A/P-CeO_2_ was also confirmed on healthy rats by determining the ratio of ceria nanoparticles in brain tissue and injected (Figure 4C). Finally, results showed that E-A/P-CeO_2_ displayed ROS scavenging ability and decreased the infarct volume on rats (Figure 4D). The results showed that ceria nanoparticles might be promising in treating I/R injury.

Platinum nanoparticles (nPt) are novel ROS scavengers because the large surface area and the high electron density can potentiate their catalytic activity to quench ROS [75]. Takamiya et al. investigated whether nPt (2–3 nm) has the neuroprotection effect against I/R injury in the mouse model of transient middle cerebral artery occlusion (tMCAO) [64]. The results showed that treatment with nPt ameliorated the generation of superoxide via reduction of hydroethidine in the cerebral cortex and decreased the mouse infarct volume. The same group published another work to investigate whether nPt could ameliorate tissue plasminogen activator (tPA)-related ischemic injury since tPA treatment may upregulate the expression of MMP-9, which exacerbates cerebral infarction through low-density lipoprotein receptor-related protein [65]. They found that nPt decreased the MMP-9 activity and ameliorated the disrupted neurovascular unit (NVU) after tMCAO. Those results further strengthened that nPt could be combined with tPA reperfusion treatment to treat ischemic stroke patients.

### 3.2. Delivery of Anti-inflammatory Reagents

Inflammation plays a central role in ischemic stroke, thus delivering anti-inflammatory drugs may be a novel strategy to treat ischemic stroke. Exosomes are endogenous vesicles made from cell membrane with cellular targeting features, therefore, they may be drug delivery tools [76,77,78]. In addition, exosomes have other properties, such as innate stability, biodegradability, low immunogenicity, and ability to cross BBB. Tian et al. used mesenchymal stromal cell (MSC)-derived exosomes conjugated with a c(RGDyK) peptide (called cRGD-Exo) to deliver curcumin to the ischemic brain [66]. Due to the high specific affinity of c(RGDyK) peptide to integrin α_v_β_3,_ highly expressed on cerebral vascular endothelial cells of ischemic tissues, cRGD-Exo was significantly accumulated in the stroke lesion. Administration of curcumin with exosomes (called cRGD-Exo-cur) suppressed the inflammatory response via the NF-κB pathway and protected the brain from ischemic injury.

Dexamethasone is an anti-inflammatory drug and can reduce proinflammatory cytokines, which prevent cellular damage in the ischemic brain [79]. Lee and co-workers incorporated dexamethasone to the hydrophobic component of a small peptide, R3V6 peptide (with a 3-arginine block and a 6-valine block), to form a stable micelle (named, R3V6-Dexa) used for gene delivery. R3V6-Dexa was loaded with a plasmid DNA, which expressed a heme oxygenase-1 (HO-1) as an antioxidant agent, and the complex of pSV-HO-1/R3V6-Dexa could target ischemic stroke tissues [67]. The authors stereotaxically injected the complex into rats and found that HO-1 expression was significantly increased. In addition, when mice were treated with the pSV-HO-1/R3V6-Dexa complex, the infarction size in the brain was significantly reduced compared to control groups.

Increased leukocyte infiltration is another factor to damage the brain in stroke. Within minutes after ischemia, adhesion molecules, such as P-selectin, are highly expressed on endothelial cells, and proinflammatory signals are rapidly generated [22]. Another strategy is to control the infiltration of neutrophils to alleviate the I/R injury in ischemic stroke. Targeting neutrophils may be a novel approach in treating ischemic stroke, but the methods in anti-neutrophil therapies and neutrophil depletion have failed in clinical studies. This failure may be associated with the complex signaling pathways and numerous receptors involved in neutrophil transmigration. For example, deactivating one receptor, such as anti-ICAM-1 therapy, may not be sufficient because neutrophil infiltration is involved with various types of surface proteins between neutrophils and endothelial cells [80,81]. Neutrophil depletion could be an effective approach, but systemic elimination of neutrophils can make the host vulnerable to bacterial and virus infections [82], and even impair other immune cells, such as natural killer cells [83].

**Figure 5:**
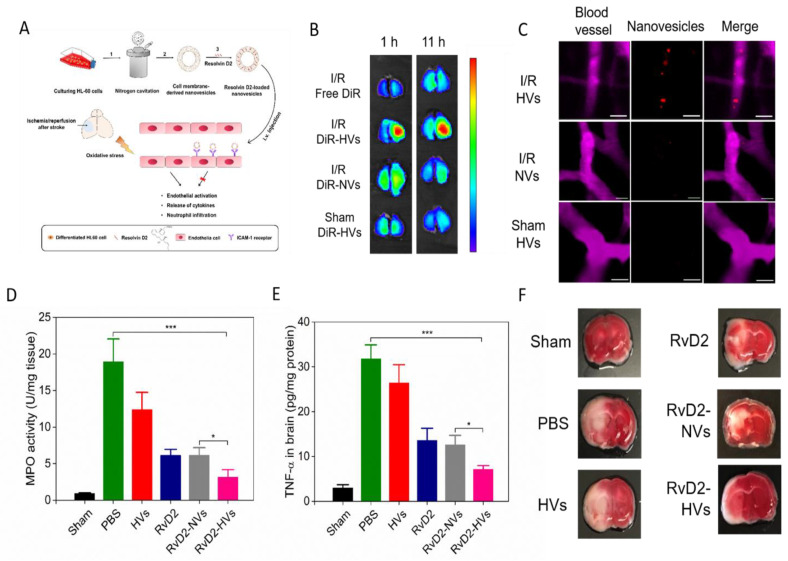
Neutrophil membrane-derived nanovesicles target ischemic endothelium and deliver therapeutics for ischemic stroke treatment. (**A**) A concept of nanovesicles binding to endothelium at ischemic/reperfusion injury sites and alleviating the I/R injury. Resolvin D2-loaded nanovesicles were prepared by: (1) nitrogen cavitation to break the cells; (2) purification; (3) Resolvin D2 loading. (**B**) Nanovesicles (HVs) specifically accumulated in I/R damaged half of brain. (**C**) Intravital microscopy images of HVs (red) specifically bind to I/R endothelium (top panel), instead of normal endothelium (bottom panel). Scale bar = 20 µm. (**D**) Myeloperoxidase (MPO) activity in the injured half of brain after different treatment, which indicates the neutrophil infiltration condition. (**E**) Level of TNF-α in the injured half of brain after different treatment. (**F**) TTC staining of brain sections in different groups. Data represent mean ± standard deviation (SD). * *p* < 0.05 and *** *p* < 0.005. Reproduced with permission [68]. Copyright 2019, American Chemical Society.

Neutrophil infiltration is regulated by interactions between neutrophils and endothelial cells. Therefore, delivering drugs to the cerebral endothelium may effectively inhibit neutrophil infiltration without interfering neutrophils [77]. Dong et al. reported neutrophil cell membrane-derived nanovesicles (HVs), which can target inflamed endothelium at I/R injury sites and deliver therapeutics to treat the mouse I/R injury [68]. The nanovesicles were generated from differentiated HL-60 cells (neutrophil-like cells) using nitrogen cavitation. The nitrogen cavitation approach was used to disrupt cell membrane to eliminate nuclei and cytosols of cells. Figure 5A shows the scheme of experimental design. The TEM image showed the liposome-like structure of nanovesicles made from HL-60 cells and the size was 200 nm in diameter. In vivo imaging system (IVIS) and confocal microscopy (Figure 5B) showed that HVs (nanovesicles made from differentiated HL-60 cells) were specifically accumulated in the injured half of the brain rather than in the normal brain. To visualize how HVs interacted with brain vasculature, a cranial window was established and intravital microscopy was performed in live mice (Figure 5C). The real-time visualization images strongly indicated that HVs can specifically target ischemic vasculature. To examine the delivery of therapeutics with nanovesicles, Resolvin D2 (RvD2) was loaded into the membrane of nanovesicles because RvD2 is a new lipid mediator to resolve inflammatory responses [84]. After Resolvin D2-loaded nanovesicles (RvD2-HVs) were intravenously injected into mice, reduced neutrophil infiltration was observed using intravital microcopy in live mice, and brain homogenates also confirmed this observation (Figure 5D). Subsequently, the level cytokines, such as TNF-α (Figure 5E), were decreased after RvD2-HVs were administered. The data indicated that RvD2-HVs could alleviate inflammation responses in ischemic stroke. The diminished inflammatory responses reduced the infarction sizes and prevented neurological damage from ischemic stroke (Figure 5F).

### 3.3. Delivery of siRNA

Small interfering RNAs (siRNAs) can suppress the expression of a specific gene and are also used in ischemic stroke treatment [85]. Caspase-3 activation contributes to brain tissue loss and downstream biochemical events, which lead to programmed cell death in many brain injuries including ischemic stroke [86]. Khuloud et al. investigated whether using carbon nanotube-mediated in vivo RNA interference (RNAi) to silence Caspase-3 could offer a therapeutic opportunity against stroke. Peri-lesional stereotactic administration of functionalized carbon nanotubes (f-CNT) carrying Caspase-3 siRNA (siCas 3) decreased the neurodegeneration, improved behaviors, and reduced ischemic lesion in an endothelin-1 induced stroke model [69].

In another study, a gene delivery vector, PAMAM-Arg, consisting of a polyamidoamine (PAMAM) dendrimer amide grafted with basic L-arginine residues (e-PAM-R), was used to deliver the high mobility group box-1 (HGMB1) siRNA [70]. HMGB1 serves as a danger signal that evokes inflammatory responses in various types of cells. After the e-PAM-R/siRNA complex was administered in the rat MCAO model, HMGB1 levels and neuronal cell death were significantly decreased. In addition, the reduction of infarction sizes in the brain was also observed.

## 4. Stroke Diagnosis using Nanotechnology

Magnetic resonance imaging (MRI), computed tomography (CT), positron emission tomography (PET), and ultrasound are employed for the diagnosis of stroke. Brain CT and MRI imaging are important to detect the early stage of stroke in patients [87]. CT angiography can discover the arterial dissection in the brain vessels and changes of collateral blood flow. Diffusion-weighted imaging is a type of MRI methods used to detect the early stage of ischemic stroke because the imaging system can localize and measure the ischemic lesion to confirm the infarct sites [88]. I/R injury is associated with vascular inflammation, so fluorescence imaging can be used to investigate the changes of brain vessels using IV injection of fluorescent dyes such as dextran- or BSA-conjugates [89].

Shen’s research groups developed a method to track administered stem cells and investigated cell-based therapies in ischemic stroke. Cationic polymersomes formed polymeric vesicles and they were loaded with superparamagnetic iron oxide nanoparticles (SPIONs) and quantum dots (QDs) as imaging agents. Cationic polymersome vesicles were incubated with neural stem cells (NSCs) to label the NSCs, thus NSCs could be tracked after they were injected to the striatum contralateral of the ischemic hemisphere. The migration and location of NSCs were monitored using MRI imaging in six weeks in a rat MCAO model and the optical imaging tracked the cells for four weeks [90]. Another example is that neural stem cells (NSCs) were labeled with an MRI reporter ferritin heavy chain (FTH) and enhanced green fluorescent protein (EGFP) to monitor the stem cells in the long-term for detecting ischemic stroke [91]. Those approaches are promising to understand how stem cells target the ischemic tissues and to develop the cell-based therapies to treat ischemic stroke.

Andreas et al. conducted a clinical phase II pilot trial using ultra small superparamagnetic iron oxide (USPIO)-enhanced MRI for macrophage imaging since USPIO particles have been introduced as a cell-specific MRI contrast agents taken up by macrophages. USPIO contrast agent was infused in ten patients 5 to 6 days after stroke onset, and MRI was performed within 24–36 h or 48–72 h after the infusion. Results showed that USPIO was much better than gadolinium (a clinically used imaging agent). The study indicates that USPIO-enhanced MRI may provide an in vivo tool to track cells in stroke and other CNS pathologies [92].

Theranostics nano-platform is a new way to combine diagnostics and therapeutics in ischemic stroke. In a study, HSP72 antibodies against HSP72, a specific molecular biomarker of the peri-infarct region, were conjugated to liposomes. Rhodamine/gadolinium labelled- and citicoline (CDP-Choline)-loaded liposomes specifically targeted the peri-infarct tissue in cerebral ischemia. MRI results demonstrated that the nanoparticles were accumulated in the injured brain and significantly reduced lesion volumes [93].

Clinically used gadolinium (Gd) chelates as contrast agents have several issues, such as short circulation time, rapid clearance, and low T1 signal. These issues limit the imaging time and resolution [94]. Upconversion nanoparticles doped with Gd ions can greatly enhance the T1 signals. In addition, upconversion nanoparticles can image the deep tissue and possess the photostability. Jing et al. reported synthetic UCNPs of core/shell structure (NaYF_4_:Yb/Er@NaGdF_4_) and coated with PEG (PEG-UCNPs). PEG-UCNPs showed the high diagnostic sensitivity to image acute ischemic stroke at a low dosage (5 mg Gd kg^−1^) compared to the clinical dosage of 108 mg Gd kg^−1^ [95]. This new formulation may be promising in clinic.

## 5. New Opportunities and Perspectives

Stroke is an acute disease, therefore treating stroke requires the early diagnosis and immediate therapies. This review has highlighted the recent advances in design and engineering of new nanomaterials to target ischemic stroke tissues and new technologies used to image and monitor the disease progression in vivo.

Specifically delivering therapeutics to the injured brain is essential in treating ischemic stroke. While many nanoparticle-based formulations or cell-based platforms have been developed, the fundamental question of how they target ischemic stroke lesion has not been clearly addressed. For example, BBB is the blood vessel barrier to prevent therapeutics across the blood vessels. Most studies showed the dramatic therapeutic effects of using nanoparticle-based formulations compared to free drugs. The enhanced outcomes claimed that nanoparticles transported therapeutics across BBB, but the direct in vivo experimental data were vague to support this conclusion. Developing advanced in vivo imaging systems [96,97] is needed to visualize the intact brain [68] and to address whether and how nanoparticles cross the BBB.

Brain vasculature during stroke shows the temporal opening, and this disruption of BBB may be a target to guide small molecules across the BBB. Further investigation is needed on the time course of BBB opening to design ideal drug delivery platforms. Although ischemic stroke increases brain vascular permeability, the endothelial gaps are unlikely to allow the efficient transport of nanoparticles because their size is usually larger than endothelial gaps. Therefore, developing new and novel concepts is needed to solve the drug delivery across BBB. Ischemic stroke and reperfusion cause acute inflammatory responses including neutrophil infiltration across blood vessels. Recent studies have shown that rational design of nanoparticles (gold nanoparticles, polymeric nanoparticles, or protein nanoparticles) enables neutrophils to transport nanoparticles across the blood vessel barrier in infection and cancer mouse models [6,98,99,100,101]. It is expected that this technology of hijacking neutrophils in vivo may transport nanotherapeutics across BBB for therapies of ischemic stroke.

Ischemic stroke is strongly correlated to inflammatory responses. Inflammatory responses include endothelial activation and neutrophil infiltration, which damages the brain tissues. Targeting activated endothelium using cell membrane-derived nanovesicles has demonstrated the value in delivering therapeutics to treat ischemic stroke [68]. To translate cell-derived nanovesicles, developing new technologies is needed to scale up their production. Recent studies show that nitrogen cavitation methods [77] and other approaches [102] could have the potential to scale up cell-derived nanovesicles for clinical applications. In addition, Dong [68] et al. reported an interesting study to deliver lipid mediators [84] (such as Resolvin D2) to treat ischemic stroke. This study is different from current therapies that mainly deliver anti-inflammatory agents. Anti-inflammatory therapies can cause side effects [103], but Resolvin D2 is a new drug to increase the host immune defense via increased neutrophil apoptosis and macrophage phagocytosis [104]. In the future, it is needed to investigate how to efficiently load lipid mediators in nanovesicles [105] for improved treatment of ischemic stroke. Another direction is the design of new nanoparticles in response to inflammatory environments (such as pH or enzymes) [8] to improve the treatment of ischemic stroke.

Targeting inflammatory neutrophils in situ to block brain neutrophil infiltration is a new opportunity to treat ischemic stroke. A recent study [7] shows that albumin protein-formed nanoparticles loaded with doxorubicin could induce neutrophil apoptosis, thus inhibiting neutrophil infiltration to prevent brain damage in a mouse ischemic stroke model. This is an exciting and new research area to develop novel therapies to solve the lacking pharmacological therapies for ischemic stroke in clinic.

In addition, developing new drugs that can target inflammatory pathways for management of the host injury during ischemic stroke is needed. The pathogenesis of ischemic stroke is complicated, and it is involved with multiple signaling pathways. The molecular mechanisms of ischemic stroke-induced brain injury are needed to be further determined. The timing of administering drugs or nanoparticle-based therapeutics is also very critical. Optimizing therapeutic windows in the future is needed.

Theranostics formulations are interesting and promising in treating ischemic stroke since they combine diagnosis and therapies. Nanoparticle-based platforms are novel constructs because they can contain imaging agents and drugs in single nanoparticle platforms. For instance, formulations with both neuroprotectants and Fe_2_O_3_ magnetic nanoparticles can achieve the therapy and imaging. In the future, developing more similar drug delivery systems is needed to treat ischemic stroke. However, considering that many inorganic materials do not naturally exist in the body (although iron oxide nanoparticles (IONPs) have been approved by the US food and drug administration (FDA) to treat anemia), fully evaluating the biodistribution and toxicity after systemic administration is required.

## 6. Conclusions

In summary, we have discussed various nanoparticle-based formulations and cell-based platforms used in treating ischemic stroke. It is essential to understand the pathogenesis of ischemic stroke and how nanoparticles interact with ischemic tissues. The fundamental question on how nanoparticles transport therapeutics across BBB is yet to be addressed, and advances in design and synthesis of nanoparticles and novel in vivo imaging systems (such as intravital microscopy) may address this question. The development of new drugs and novel nanoparticle-based therapeutics will improve the outcomes in treating ischemic stroke patients.

## Figures and Tables

**Table 1:** Studies of nanoparticle-based drug delivery in ischemic stroke.

Nanoparticles	Drugs/agents	Administration	Targeting	Stoke model	Results	Ref.
PEGylated liposomes	Asialo-erythropoietin	i.v.	Ischemic site	MCAO (1 h) * on rats	PEGylated liposomes increased the accumulation of AEPO at ischemia site and ameliorated cerebral I/R injury.	[48]
T7-conjugated liposomes	ZL006	i.v.	TfR over-expressed brain endothelium	MCAO (2 h) * on rats	T7 enhanced the transport of liposomes across the BBB and T7-PLPs/ZL006 reduced infarct volume and neurological deficit.	[49]
SHp and T7 conjugated-PEGylated liposomes	ZL006	i.v.	Ischemic endothelium and neurons	MCAO (2 h) * on rats	Dual targeting peptide enhanced the accumulation of NPs in brain and ameliorated ischemic injury.	[50]
Liposomes	Basic fibroblast growth factor (bFGF)	Intranasal injection	-	MCAO (2 h) * on rats	Liposomes improved bFGF accumulation in brain tissues and the system improved spontaneous locomotor activity of animals.	[51]
PEGylated liposomes	t-PA/fasudil	i.v.	-	MCAO (2 h) * on rats	Treatment of fasudil-lip before t-PA decreased the risk of t-PA-derived cerebral hemorrhage and extended the therapeutic time window of t-PA.	[52]
DSPE-PEG2000 liposome	-	i.v.	Ischemic site	MCAO (20 min) * on mice	Liposomes accumulated in ischemic brain at both early stage (0.5) and late phase (48 h).	[53]
Cationic bovine serum albumin-conjugated PEG-PLA	Tanshinone IIA	i.v.	Brain microvessels	MCAO (2 h) * on rats	CBSA-PEG-TIIA-NPs reduced infarct volume and neurological deficits.	[54]
Cationic bovine serum albumin-conjugated PEG-PLA	Tanshinone IIA	i.v.	Brain microvessels	MCAO (2 h) * on rats	CBSA-PEG-TIIA-NPs suppressed neuronal apoptosis and inflammatory mediators (MMP-9 and COX-2) via MAPK signaling pathways.	[55]
SHp conjugated, red blood cell membrane shelled-polymer	NR2B9C	i.v.	Ischemic neurons	MCAO (2 h) * on rats	SHp-RBC-NP targeted to the ischemic site and ameliorate neuroscores and infarct volume.	[56]
Fas ligand conjugated-PEGylated-lipid NPs	3-n-Butylphthalide	i.v.	Microglia cells in ischaemic region	MCAO (45 min) on mice	By the help of Fas ligand, the NPs reached to ipsilateral region of ischemic brain and delivered the drug to improve brain injury.	[57]
AMD3100-conjugated PEG-T/M-PCL block copolymers (ASNPs)	Glyburide	i.v.	CXCR4 in the ischemic brain	MCAO on mice	ASNPs penetrated the ischemic brain and improve the neurological outcomes after ischemia.	[58]
TfRMAb-conjugated PEG-coated chitosan nanospheres	Z-DEVD-FMK, caspase-3 inhibitor	i.v.	TfR type 1 on the cerebral vasculature	MCAO (2 h) * on mice	Nano-system inhibited caspase activity and had subsequent neuroprotection effect.	[59]
T7-conjugated erythrocyte-coated Mn_3_O_4_ NPs	-	i.v.	Ischemic endothelium	MCAO (0.5 h) * on rats	The NPs scavenged free radical and restored the oxygen.	[60]
Platelets coated-γ-Fe_2_O_3_ magnetic NPs	L-arginine	i.v.	Thrombus	Photochemical-induced thrombosis	L-arginine released at ischemic lesions disrupted the local platelets aggregation and recover blood flow.	[61]
PEGylated-ceria nanoparticles	-	i.v.	-	MCAO on rats	Ceria nanoparticles served as ROS scavenger and reduced ischemic brain damage.	[62]
Angiopep-2 and PEG modified Ceria nanoparticles	Edaravone	i.v.	LRP on BBB	MCAO on rats	E-A/P-CeO2 crossed BBB and delivered Edaravone to eliminate ROS, protected the BBB integrity and ameliorated ischemic injury.	[63]
Platinum nanoparticle (nPt)	-	i.v.	-	MCAO (1 h) * on mice	Platinum nanoparticle reduced ROS production and improved the neurological score.	[64]
Platinum nanoparticle (nPt)	-	i.v.	-	MCAO (1 h) * on mice	nPt protected the ischemic brain via reducing the MMP-9 activity and disruption of neurovascular unit.	[65]
c(RGDyK) peptide- conjugated exosomes	Curcumin	Stereotaxic injection	Ischemic site	MCAO (1 h)* on mice	They targeted the lesion region of the ischemic brain and entered microglia to suppress the inflammatory response and protect ischemic brain.	[66]
R3V6 peptides	Dexamethasone and heme oxygenase-1 siRNA	i.v.	-	MCAO (1 h) * on rats	Dexamethasone enhanced the delivery ability of R3V6 peptides for heme oxygenase-1 (HO-1) gene knockdown and reduced ischemic brain damage.	[67]
Neutrophil membrane-derived nanovesicles	Resolvin D2	i.v.	Inflamed endothelium	MCAO (1 h) * on mice	Nanovesicles specifically targeted the ischemic endothelium and released Resolvin D2 to inhibit neutrophil infiltration and reduce inflammation in ischemic sites.	[68]
Functionalized carbon nanotubes (f-CNT)	Caspase-3 siRNA	Stereotaxic injection	Ischemic neurons	Endothelin Stroke Model	Gene silencing of Caspase-3 by f-CNT in neuronal tissue had neuroprotection effect and improved ischemic insult.	[69]
PAMAM dendrimer amide with basic L-arginine residues	HMGB1 siRNA	i.v.	HMGB1 mRNA in brain	MCAO (1 h) * on rats	HMGB1 siRNA delivered by e-PAM-R reduced neuronal death decreased infarct volume.	[70]

* The times represent the ischemic duration; (-) denotes that the information is not available in the studies.
